# Low prevalence of hepatitis C infection among HIV-infected individuals in Slovenia: a nationwide study, 1985–2013

**DOI:** 10.1186/1471-2334-14-S4-O15

**Published:** 2014-05-29

**Authors:** Katja Seme, Mateja Škamperle, Maja M Lunar, Polona Maver Vodičar, Janez Tomažič, Ludvik Vidmar, Primož Karner, Tomaž Vovko, Blaž Pečavar, Mojca Matičič, Mario Poljak

**Affiliations:** 1 Institute of Microbiology and Immunology, Faculty of Medicine, University of Ljubljana, Slovenia; 2 Department of Infectious Diseases, University Medical Center, Ljubljana, Slovenia

## 

After introduction of highly active antiretroviral therapy and consecutive successful control of HIV infection hepatitis C virus (HCV) has become an important pathogen in HIV infected patients. HIV infection in a person who is also HCV infected results in reduced rate of spontaneous HCV RNA clearance, faster liver disease progression and more aggressive course of liver disease.

A total of 639 individuals were cumulatively reported as HIV-infected in Slovenia until the end of 2013. The majority of HIV-infected were men (553/639; 86.5%) and among them 68.2% were men who have sex with men. The predominant HIV-1 subtype in Slovenia is subtype B, which is present in 85.8% of the infected individuals.

We tested 575 (90.0%) of 639 Slovenian individuals who were confirmed as HIV positive by the end of 2013 for HCV infection. All individuals included in a study were tested for both anti-HCV and HCV RNA. Out of 575 HIV-infected individuals 44 (7.6%) had anti-HCV specific antibodies, and 32 of them (72.7%) were also HCV RNA positive. We didn’t detect HCV RNA alone in any of the 531 anti-HCV-negative individuals. Anti-HCV positivity was significantly more frequent in HIV-infected individuals who acquired HIV by parenteral route (73.3%) comparing with those who acquired HIV by sexual route (2.6%). The most prevalent HCV genotype among HIV-infected individuals was genotype 1 (70.8%), followed by genotype 3 (16.7%), genotype 4 (8.3%) and genotype 2 (4.2%). HCV genotypes distribution didn’t significantly differ between HIV-positive and HIV-negative, HCV-positive Slovenian patients.

Our study which was performed on the highest proportion per entire population of HIV-infected individuals from a certain country identified Slovenia as the country with the lowest prevalence of HCV infection among HIV-infected individuals. The predominance of sexual transmission of HIV (79.2%) in Slovenia and the fact that HIV has not yet entered the intravenous drug users’ community in Slovenia are the two most likely reasons for low prevalence of HIV-HCV co-infection. However, the present epidemiological situation in Slovenia needs to be monitored closely since it could quickly change in a case of an increase in the incidence of acute hepatitis C among HIV-infected men who have sex with men and/or increase of HIV infection among intravenous drug users in the country, as it happened recently in some neighboring countries.

